# Detection of YAP1 and AR-V7 mRNA for Prostate Cancer Prognosis Using an ISFET Lab-On-Chip Platform

**DOI:** 10.1021/acssensors.2c01463

**Published:** 2022-11-11

**Authors:** Joseph Broomfield, Melpomeni Kalofonou, Thomas Pataillot-Meakin, Sue M. Powell, Rayzel C. Fernandes, Nicolas Moser, Charlotte L. Bevan, Pantelis Georgiou

**Affiliations:** † Centre for Bio-Inspired Technology, Department of Electrical and Electronic Engineering, Imperial College London, LondonSW7 2AZ, U.K.; ‡ Imperial Centre for Translational and Experimental Medicine, Department of Surgery and Cancer, Imperial College London, LondonW12 0NN, U.K.; § Sir Michael Uren Hub, Department of Bioengineering, Imperial College London, LondonW12 0BZ, U.K.; ∥ Molecular Science Research Hub, Department of Chemistry, Imperial College London, LondonW12 0BZ, U.K.

**Keywords:** prostate cancer, lab-on-chip, point-of-care device, RT-LAMP, AR-V7 and YAP1 RNA, ISFETs, sensors

## Abstract

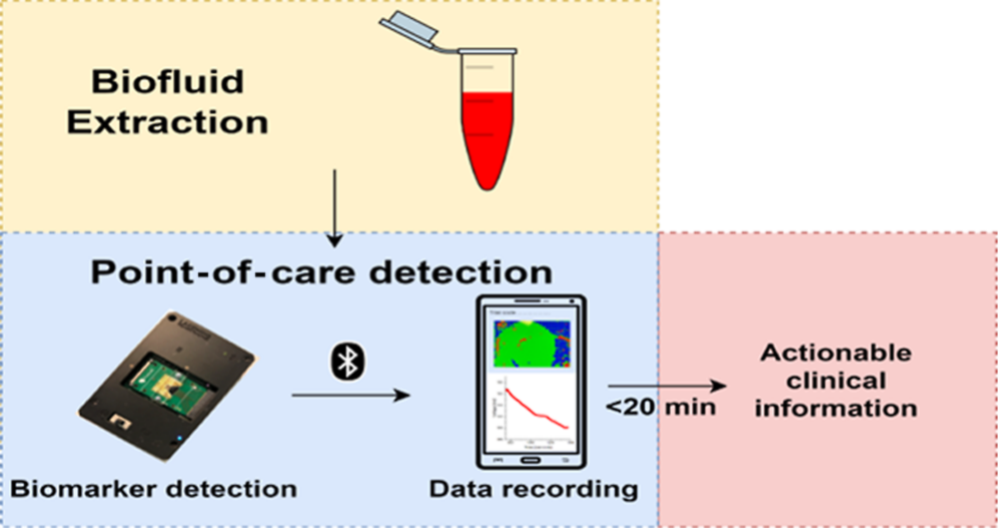

Prostate cancer (PCa) is the second most common cause of male cancer-related death worldwide. The gold standard of treatment for advanced PCa is androgen deprivation therapy (ADT). However, eventual failure of ADT is common and leads to lethal metastatic castration-resistant PCa. As such, the detection of relevant biomarkers in the blood for drug resistance in metastatic castration-resistant PCa patients could lead to personalized treatment options. mRNA detection is often limited by the low specificity of qPCR assays which are restricted to specialized laboratories. Here, we present a novel reverse-transcription loop-mediated isothermal amplification assay and have demonstrated its capability for sensitive detection of AR-V7 and YAP1 RNA (3 × 10^1^ RNA copies per reaction). This work presents a foundation for the detection of circulating mRNA in PCa on a non-invasive lab-on-chip device for use at the point-of-care. This technique was implemented onto a lab-on-chip platform integrating an array of chemical sensors (ion-sensitive field-effect transistors) for real-time detection of RNA. Detection of RNA presence was achieved through the translation of chemical signals into electrical readouts. Validation of this technique was conducted with rapid detection (<15 min) of extracted RNA from prostate cancer cell lines 22Rv1s and DU145s.

## Introduction

One in eight men are expected to be diagnosed with prostate cancer (PCa) within their lifetime.^1^ Aggressive tumors progress to metastatic castration-resistant prostate cancer (mCRPC) which is responsible for the majority of PCa-related deaths.^2^ Other patients, however, will have clinically insignificant PCa, where the longevity and quality of a patient’s life is not adversely affected by PCa presence.^3^ Successfully determining between aggressive and clinically insignificant PCa is crucial to affording patients’ appropriate treatment. Current clinical diagnosis for PCa relies on multi-parametric MRI, PSA testing, and trans-rectal ultrasound-guided biopsy.^4^ PSA screening in the UK is not currently implemented based on the limited benefits at diagnosing PCa on account of false negatives and false positives.^5^ Current testing for PCa is very limited prognostically and often leads to overtreatment of patients with clinically insignificant PCa. Another urgent biomarker requirement is for the accurate and early detection of resistance to hormonal therapies, that is, the development of castration resistance. This would facilitate the prompt discontinuation of ineffective therapies (with their significant side effects) and potential adoption of new approaches.

Recent research has indicated that detection of circulating biomarkers including cell-free DNA, microRNAs, mRNAs, and circulating tumor cells present a minimally invasive alternative to current testing methods.^6−9^ However, RNA and DNA detection is often compounded by the limited specificity of qPCR assays.^10,11^ In addition, the relative low abundance in circulating biofluids of mRNA and its inherent lability can make this species a challenging yet potentially valuable dynamic biomarker for PCa prognosis. Detection of mRNA biomarkers at the point-of-care (PoC) could provide rapid *in situ* responses to direct treatment options for PCa patients. Previous work has established several mRNAs of interest for PCa prognostics, including both androgen receptor (AR) variant 7 (AR-V7) and Yes-associated protein 1 (YAP1) mRNA.^12,13^ AR-V7 is deficient of the ligand binding domain (LBD) which normally makes the AR a ligand-activated transcription factor, as a result it is constitutively active. As such, AR-V7 presence in PCa patients is often associated with resistance to androgen deprivation therapy (ADT), the gold standard treatment for disseminated disease which targets the AR LBD.^14^ Across data extracted from 12 clinical trials, the proportion of mCRPC patients with detectable circulating AR-V7 mRNA is 18.3%.^15^ Detection of circulating AR-V7 mRNA in mCRPC patients treated with ADT, corresponded to reduced overall survival and progression free survival in these patients, supporting AR-V7 as clinically actionable mRNA for detection in the blood.^12^ YAP1 has multiple roles, including as a mechanosensor. Stiff matrices result in nuclear localization of YAP1 where transcriptional regulation for cell survival and proliferation can take place.^16^ As such, YAP1 is commonly associated with the epithelial to mesenchymal transition in several types of cancers.^17−20^ YAP1 upregulation in the nucleus is correlated with reduced overall and disease-free survival in various cancers.^21−23^ In multiple PCa cell lines, YAP1 knockdown is associated with reduction in cellular motility, invasion, and progression to metastatic phenotypes.^24−26^ However, the *YAP1* gene is downregulated by late stage PCa-associated miR, miR-375-3p in mCRPC samples.^13,20^ Therefore, YAP1 potentially presents a temporal biomarker for progression from locally advanced PCa to mCRPC. Because the miR-375-3p - YAP1 pathway is implicated in docetaxel resistance, it could also direct treatment for mCRPC patients.^13^

qPCR is commonly referred to as the “gold standard” for nucleic acid amplification tests, on account of its high accuracy and sensitivity. However, thermal cycling equipment crucial to qPCR experimentation is expensive and limited to use in specialized laboratories.^27^ As a result, qPCR experimentation is further compounded by transfer times to a laboratory. Alternative solutions for amplification tests are therefore required for PoC prognostic and diagnostic tests. Loop-mediated isothermal amplification (LAMP), developed and optimized by Notomi et al. and Nagamine et al., respectively, is a rapid (<30 min) and sensitive DNA amplification technique.^28,29^ LAMP utilizes six primers targeting eight specific DNA regions for exponential and isothermal amplification resulting in a high-yielding DNA assay.^28^ Reverse transcriptase LAMP (RT-LAMP) allows application of the technique to mRNA and has previously been used to detect mRNA in various diseases, including distinguishing dengue serotypes, prostate cancer antigen 3 for PCa diagnosis, and more recently the N gene for SARS-CoV-2 virus detection.^30−32^ Integration of LAMP assays with ion-sensitive field-effect transistors (ISFETs) and unmodified complementary metal oxide semiconductor technology for lab-on-chip (LoC) detection of biomarkers has previously been successful.^32−36^ RT-LAMP can be adjusted to result in a pH readout (RT-pHLAMP) during amplification events (i.e., a positive signal), which allows for compatibility with the pH-sensing ISFET for use in a microfluidic PoC device.^37,38^ Double-stranded DNA synthesis, which occurs in the RT-pHLAMP amplification event, releases a proton per nucleotide addition to the DNA strand.^39,40^

**Figure 1:**
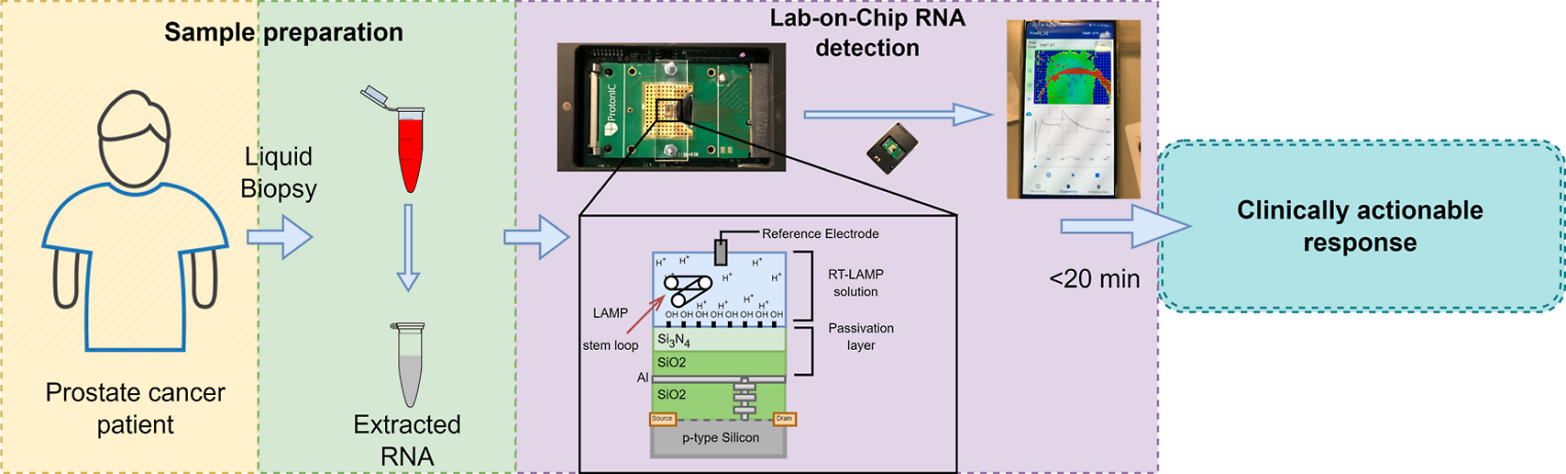
Prospective workflow from liquid biopsy extraction from a PCa patient to a clinically actionable response via mRNA detection using an ISFET biosensor and an optimized RT-LAMP assay.

This work presents a method with bespoke primer selection and optimization for the *de novo* development of RT-LAMP assays for the detection of AR-V7 and YAP1 mRNA. Adaptation of this assay for ISFET compatibility resulted in an accurate, sensitive (3 × 10^1^ copies per reaction), and rapid (<15 min) test for YAP1 and AR-V7 synthetic RNA presence. The assays were successfully tested on the ISFET LoC device presenting use of this device for PoC. Validation of this assay and the LoC device was confirmed with detection of AR-V7 and YAP1 mRNA extracted from PCa cell lines. The development of this biosensor and these assays present the potential for PoC prognostics, where clinicians can rapidly adjust treatment options for PCa patients (Figure 1). Although the rapidity of the device is unlikely to be essential for PCa prognosis, the potential for an accurate and cost-efficient handheld device requiring non-specialized personnel would be of significant benefit.

## Results

### RT-qLAMP and RT-pHLAMP Assay Optimization for AR-V7 and YAP1 Detection

**Figure 2:**
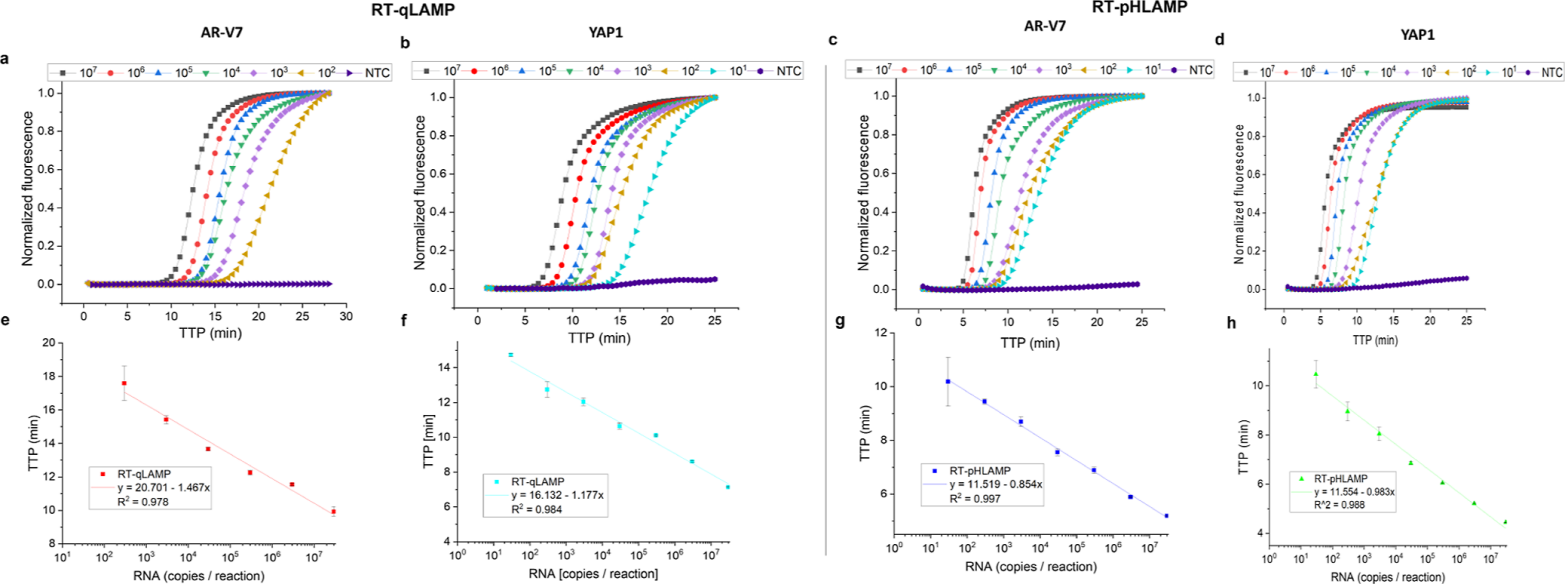
(a–d) Sigmoidal amplification curves of RT-qLAMP and RT-pHLAMP assays detecting AR-V7 and YAP1 synthetic RNA. Synthetic RNA concentrations varied from 3 × 10^7^ to 3 × 10^2^ copies per reaction for the AR-V7 RT-qLAMP reaction and 3 × 10^7^ to 3 × 10^1^ copies per reaction for each RT-pHLAMP reaction and the YAP1 RT-qLAMP reaction. Data are averaged across two experiments. (a) Amplification curve of the RT-qLAMP assay detecting synthetic AR-V7 RNA. (b) Amplification curve of the RT-qLAMP assay detecting synthetic YAP1 RNA. (c) Amplification curve of the RT-pHLAMP assay detecting synthetic AR-V7 RNA. (d) Amplification curve of the RT-pHLAMP assay detecting synthetic YAP1 RNA. (e–h) Standard curves of RT-qLAMP and RT-pHLAMP detection of synthetic AR-V7 and YAP1 RNA at varying concentrations. These graphs include linear regressions, the coefficient of determinations of each assay, and error bars displaying one standard deviation. (e) Standard curve of the RT-qLAMP assay detecting synthetic AR-V7 RNA. (f) Standard curve of the RT-qLAMP assay detecting synthetic YAP1 RNA. (g) Standard curve of the RT-pHLAMP assay detecting synthetic AR-V7 RNA. (h) Standard curve of the RT-pHLAMP assay detecting synthetic YAP1 RNA.

Initial optimization of the RT-qLAMP assay rendered the primers as presented in Table 1.^41^ Different lengths of the front inner primer and back inner primer were tested to ensure that optimal time to positive (TTP) values were achieved. The AR-V7 primers specifically targeted a region in cryptic exon 3 to avoid amplification of the full-length androgen receptor (AR-FL) mRNA.^42^ Recent evidence has suggested that AR-V7 cryptic exon mRNA in the blood is more abundant than mRNA across splice boundaries, further supporting the target region for primer design.^43^ Because mRNAs present in the blood are often fragmented, synthetic RNA fragments of both AR-V7 and YAP1 target regions (374 and 355 bp lengths, respectively) were synthesized for initial assay development.^44,45^ Both the AR-V7 and YAP1 RT-qLAMP assays achieved linear detection of 3 × 10^7^ to 3 × 10^2^ copies of synthetic RNA per reaction in under 18 min (Figure 2). The YAP1 RT-qLAMP assay showed a greater quantitative detection limit down to 3 × 10^1^ copies per reaction.

**Table 1:** Primer Sequences for Both AR-V7 and YAP1 RT-qLAMP and RT-pHLAMP Assays

AR-V7 RT-qLAMP primers	sequence 5′ → 3′	YAP1 RT-qLAMP primers	sequence 5′ → 3′
V7 F3	CTAGCCTTCTGGATCCCA	YAP1 F3	TTTGCCCAGTTATACCTCA
V7 B3	AGGCTAGATGTAAGAGGGA	YAP1 B3	CAAGAAGCAGTTAAGCACTT
V7 FIP	TTCTGTGGATCAGCTACTAACCTAGA	YAP1 FIP	TCAGTACAGAGGGCATCGTTAGCAGT
	TCTTAGCCTCAG		ACTGTGATACCT
V7 BIP	AGTAAACAAGGACCAGATTTCTGTAG	YAP1 BIP	CCTGAAGGAGACCTAAGAGTCAGGAC
	TCTCTCAGTGTGTTTGA		ATAAAACAAGAGACCA
V7 LF	GCTCAGTGACAGGGCCTGAG	YAP1 LF	CAAAGCACTGTGCCAGGT
V7 LB	CCAGGAGAAGAAGCCAGCCA	YAP1 LB	CCCTTTTTGAGTTTGAATCATAGCC

In order to generate a pH readout for ISFET compatibility, the RT-qLAMP assays were adjusted as previously described to omit tris(hydroxymethyl)-aminomethane (tris), the pH buffering agent present in Isothermal Amplification Buffer (New England Biolabs).^37^ Betaine was further omitted in the augmented assay to equate for lyophilization compatibility. The resulting RT-pHLAMP assays subsequently showed a sensitivity of 3 × 10^1^ RNA copies per each reaction (Figure 2). The standard curves of these reactions presented coefficients of determination (*R*^2^) of 0.997 and 0.988 for the AR-V7 and YAP1 RT-pHLAMP assays, respectively, which indicates the potential of these assays for accurate quantification of RNA per sample. TTP values for the pH sensitive reactions were significantly reduced: the average TTP for detection of 3 × 10^2^ copies of synthetic AR-V7 RNA was 17.6 min in RT-qLAMP and 9.5 min in RT-pHLAMP. This is likely due to the increased optimization of the RT-pHLAMP assay, allowing for faster TTP values. Detection from 3 × 10^7^ to 3 × 10^1^ copies of RNA was achieved in under 12 min for both RT-pHLAMP assays.

Specificity of the AR-V7 RT-pHLAMP reaction was confirmed by spiking the assays with a synthetic RNA fragment present in the AR-FL LBD (Supporting Information, Figure S4). Primers detecting this AR-FL region were developed to confirm its presence in these spiked assays (Supporting Information, Figure S5). No amplification occurred between the AR-FL synthetic RNA and AR-V7 primers after the reaction was terminated at 35 min. A serial dilution experiment for AR-V7 detection spiked with AR-FL then took place. These results indicate that amplification of the AR-V7 RT-pHLAMP assay only occurred with the presence of AR-V7 mRNA. In this instance, the sensitivity of the reaction was reduced to 3 × 10^2^ copies, indicating that the presence of off-target RNA decreased the efficiency of the RT-pHLAMP assay.

### Validation of AR-V7 and YAP1 RT-pHLAMP Specificity with Extracted RNA from Prostate Cancer Cell Lines

**Figure 3:**
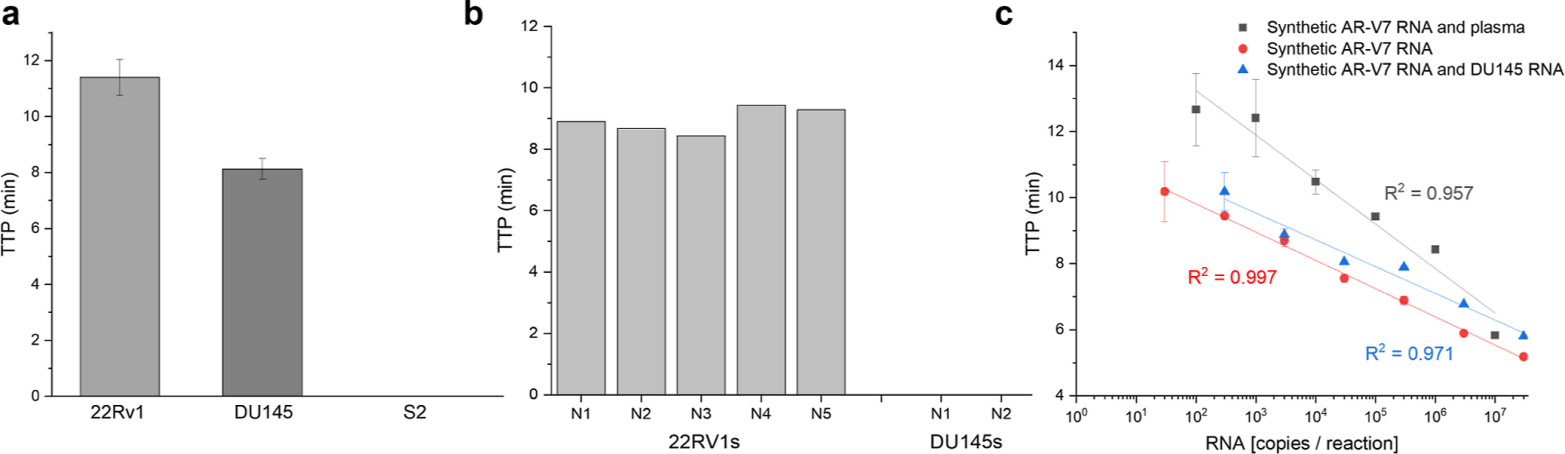
(a) Variation in TTP in the YAP1 RT-pHLAMP assay with 1 ng of extracted mRNA from two PCa cell lines, DU145s and 22Rv1s, and S2 RNA from *D. melanogaster*. (b) Variation in TTP in the AR-V7 RT-pHLAMP assay with 1 ng of extracted RNA from AR-V7 positive cell line, 22Rv1, and the AR-V7 negative cell line, DU145. (c) Standard curves for multiple AR-V7 RT-pHLAMP experiments including the unmodified synthetic AR-V7 mRNA assay, the assay spiked with off-target DU145 mRNA, and the assay containing human plasma.

Extracted RNA from PCa cell lines 22Rv1 and DU145 was utilized to confirm the detection of endogenous YAP1 and AR-V7 mRNA. 22Rv1s have previously been reported as AR-V7 mRNA positive while DU145s show little to no AR-V7 expression.^46^ Five individual 22Rv1 RNA samples rendered an average TTP of 8.17 ± 0.54 min with 1 ng of RNA per reaction (Figure 3b). In contrast, 1 ng per reaction of extracted RNA from DU145s rendered no fluorescent signal after 35 min, indicating no amplification had taken place. These findings suggest the AR-V7 RT-pHLAMP assay is specific to AR-V7 mRNA in patient-derived cell lines.

In order to determine if off-target RNA affected the efficiency of the RT-pHLAMP assay, synthetic AR-V7 mRNA was spiked with 1 ng of DU145 mRNA. A serial dilution experiment was then conducted (Figure 3c). These results suggest that the presence of off-target RNA marginally increased the TTP values at various concentrations in the AR-V7 RT-pHLAMP assay. In addition, reliable and quantitative detection of synthetic RNA was achieved down to 3 × 10^2^ copies per reaction. Utilizing the standard curve generated from this experiment, the average copy number of AR-V7 mRNA per 1 ng of RNA is 1.6 × 10^4^ copies.

To indicate if both the RT-pHLAMP assays for AR-V7 and YAP1 mRNA were feasible for detection of circulating mRNA in the blood, assays including citrated human plasma were conducted (Figure 3c and Supporting Information, Figure S9, respectively). The limit of detection in these experiments was 1 × 10^2^ copies per reaction although TTP values were marginally increased. Sensitive and expeditious detection of both YAP1 and AR-V7 mRNA was therefore achieved in human plasma samples. However, pH values for these reactions indicated that no pH change took place, likely due to the carbonic acid/bicarbonate buffer system present in the blood.^47^ Integration of plasma samples directly onto the LoC platform would subsequently require further optimization, outside of the scope of this study.

YAP1 mRNA presence was also tested in RNA extracted from 22Rv1 and DU145 cell lines. High expression of YAP1 mRNA concentration has previously been recorded in DU145 cells.^24^ The RT-pHLAMP assay detected YAP1 mRNA presence in 8.08 ± 0.41 min at 1 ng per reaction across RNA extracted from two DU145 cell line samples. YAP1 presence was additionally detected in 22Rv1 RNA samples, at an increased TTP of 11.7 ± 0.68 min. miR-375 is highly expressed in 22Rv1 cell lines and targets YAP1 mRNA, resulting in its downregulation.^20^ As such, the variation in TTP values for 22Rv1 and DU145 extracted RNA samples corresponds to the expected concentrations of YAP1 mRNA in these cell lines. Welch’s unequal variances *t*-test was used to confirm the significance of this data (*t* = 14.47, *p* < 0.001). RT-qPCR assays (Supporting Information, Figure S7) confirmed the high concentration of YAP1 mRNAs in DU145s and lower concentration in 22Rv1s (*t* = 8.15, *p* < 0.001). A negative cell line for YAP1 mRNA was also introduced to confirm the specificity of the RT-pHLAMP reaction. Because endogenous expression of YAP1 is present in many human cell lines, Schneider 2 (S2) cell RNA from *Drosophila melanogaster* was utilized. Figure 3 indicates that no amplification took place in this cell line RNA with the YAP1 RT-pHLAMP assay. Similar to AR-V7, S2 cell RNA was spiked with synthetic YAP1 RNA and a standard curve was produced (Supporting Information, Figure S9). From the standard curve generated DU145s contain 5.5 × 10^3^ copies of YAP1 per 1 ng of RNA. However, because the TTP for 22Rv1 is outside of the quantitative range of the standard curve, YAP1 copy number was not ascertained for this cell line.

As expected, no amplification curves were seen in DU145s with the AR-V7 RT-qPCR assay, whereas fast amplification was observed in the 22Rv1 cell line (Supporting Information, Figure S6). This indicates that the RT-pHLAMP assay data correspond well with the gold standard of nucleic acid amplification tests.

### Implementation of AR-V7 and YAP1 RT-pHLAMP Assays onto the Lab-On-Chip Platform

The developed RT-pHLAMP assays were subsequently integrated into the lab-on-chip which utilized ISFET sensors to detect the rate of pH change. Double-stranded DNA synthesis, which occurs during the RT-LAMP amplification event (in positive samples), releases a proton per each nucleotide addition.^39,40^ The subsequent change in pH of the unbuffered RT-pHLAMP solution is detected by the ISFET and recorded by a mobile phone.

**Figure 4:**
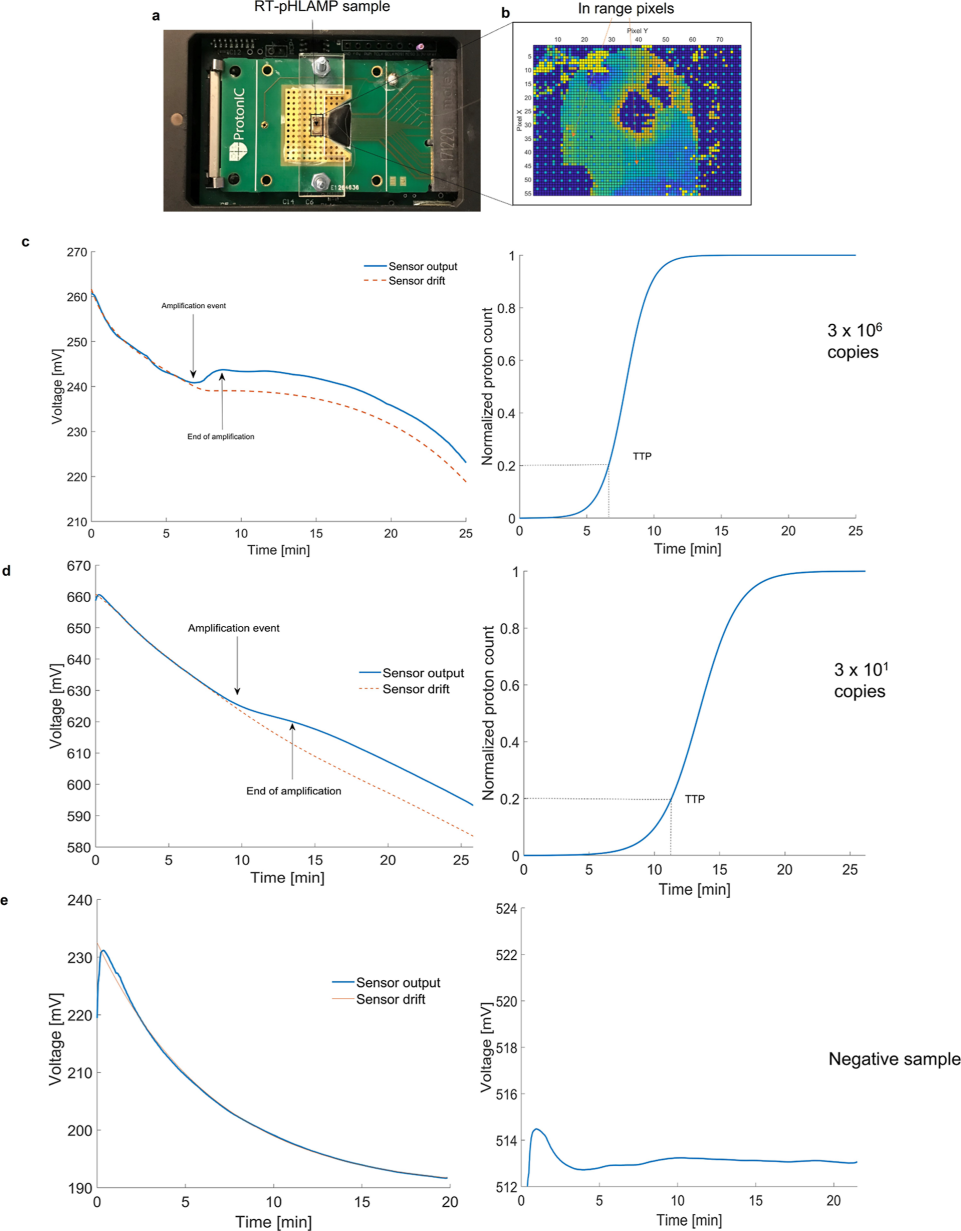
Illustration of RT-pHLAMP implementation onto the LoC platform. (a) ISFET microchip setup with an acrylic manifold and RT-pHLAMP sample loaded onto the microchip. (b) Array of the ISFET microchip once the experiment was initiated. In range pixels are shown in green/light blue. Dark blue and red indicate pixels that are out of range for pH detection. (c) ISFET sensor output graph (left) and sigmoidal-fitted amplification curve (right) of a positive AR-v7 sample on the ISFET microchip (3 × 10^6^ copies per reaction). (d) Detection of 3 × 10^1^ copies of synthetic AR-v7 RNA with the ISFET biosensor. The ISFET biosensor output graph (left) and the amplification curve with sigmoidal fitting (right) are shown here. (e) ISFET sensor output graph (left) and amplification curve (right) of a negative AR-v7 sample on the ISFET microchip. No sigmoidal fitting was performed for this experiment on account of the negative signal.

Synthetic YAP1 and AR-V7 RNA samples were successfully detected at a concentration of 3 × 10^6^ copies per reaction. TTP values were slightly increased on the LoC platform, likely due to non-optimal conditions for the RT-pHLAMP assay in the acrylic reaction chamber. These increased values are still significantly reduced (indicating more rapid detection) relative to the PCR gold standard for nucleic acid amplification tests. The averaged TTP value across triplicate experiments for detection of YAP1 and AR-V7 synthetic RNA at 3 × 10^6^ copies per reaction was 7.25 ± 0.62 min and 7.11 ± 0.65 min, respectively. Figure 4 shows the implementation of the AR-V7 RT-pHLAMP assay onto the microchip. Post-processing of the voltage readout is required to subtract the inherent drift present in ISFET biosensors.^48^ The voltage output is converted to proton count and sigmoidal fitting is then carried out to return the amplification curve illustrated in Figure 4. Conversion of the voltage output to proton count is described in Supporting Information, eqs S1 and S2.

Once the detection of 3 × 10^6^ copies of both AR-V7 and YAP1 synthetic RNA had taken place with the LoC device, the limit of detection was tested at 3 × 10^1^ copies. For both of the RT-pHLAMP assays, the pH change at 3 × 10^6^ and 3 × 10^1^ copies were similar, likely due to the DNA production being the same at both concentrations. Figure 4 additionally shows the amplification of 3 × 10^1^ copies of AR-V7 synthetic mRNA on the LoC device. Here, the TTP values were 10.88 ± 0.95 min for the AR-V7 RT-pHLAMP assay and 11.50 ± 0.98 for the YAP1 RT-pHLAMP assay. This illustrates that the sensitivity of the LoC device is comparable to the benchtop RT-pHLAMP assays.

### Detection of YAP1 and AR-V7 mRNA from Patient-Derived Cell Lines on the Lab-On-Chip Platform

**Figure 5:**
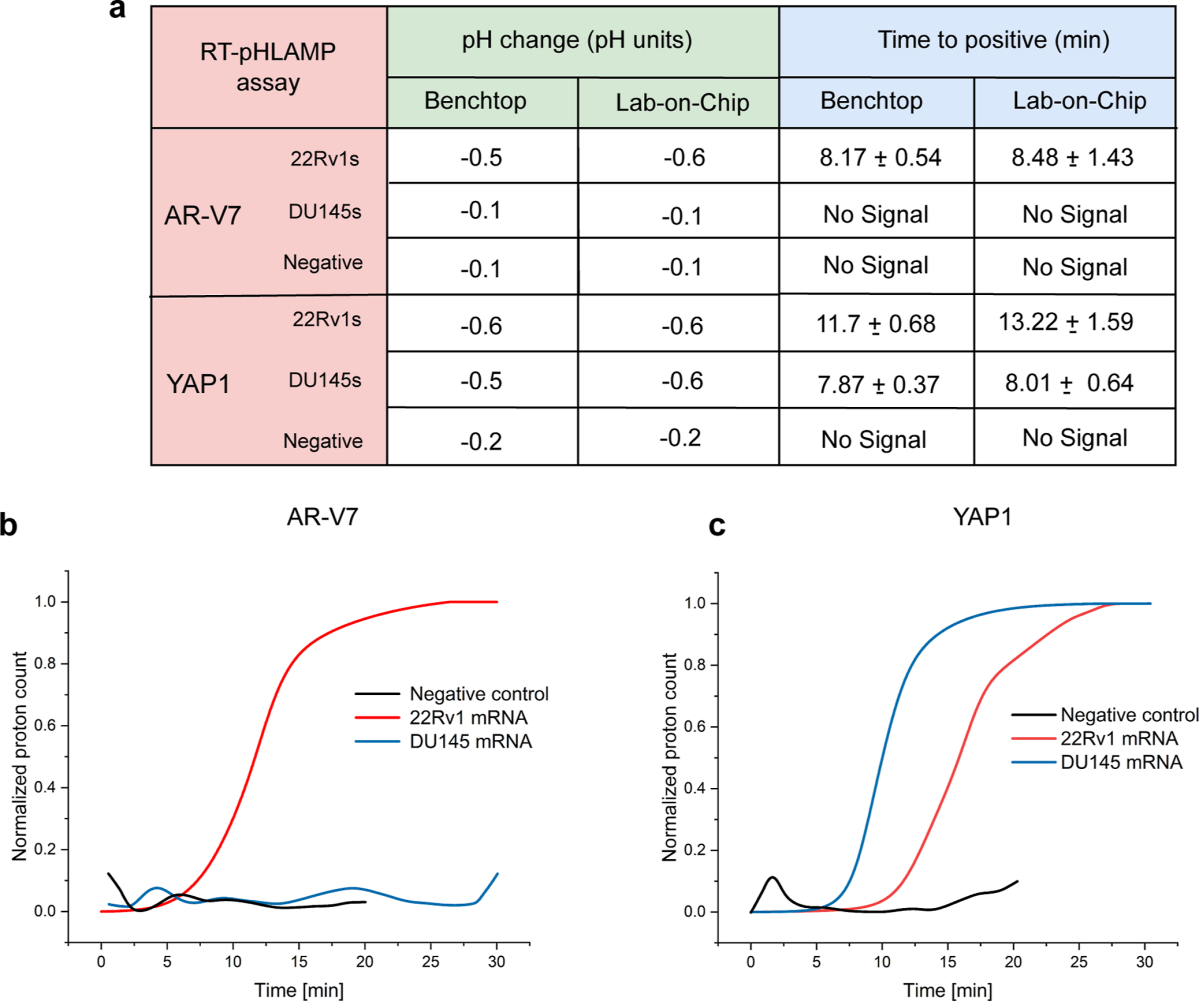
(a) TTP and pH change values of the benchtop and LoC RT-pHLAMP assays. Benchtop assays were terminated after 35 min, LoC positives were terminated after 30 min and LoC negatives after 20 min. (b) Sigmoidal-fitted averages of AR-V7 mRNA in 22Rv1s, DU145s, and negative samples using the RT-pHLAMP assay. Graphs are the average values of triplicate assays. (c) Sigmoidal-fitted averages of YAP1 mRNA in 22Rv1s, DU145s, and negative samples using the RT-pHLAMP assay. Graphs represent the average values of triplicate assays.

Once it had been determined that these assays were compatible with the ISFET biosensor, detection of AR-V7 and YAP1 mRNA present in RNA extracted from 22Rv1 and DU145 cells was assessed. As confirmed in the previous benchtop RT-pHLAMP and RT-qPCR assays, 22Rv1 cells are AR-V7 positive and DU145s contain high levels of YAP1. Figure 5 shows the ISFET detection of AR-V7 and YAP1 in the two cell lines. Here, no positive signal is detected for AR-V7 in the DU145 cell line, mirroring the expression shown in the RT-qPCR-based assay and the relevant literature.^46^ Contrastingly, detection of 1 ng of AR-V7 mRNA per reaction was achieved in 8.48 ± 1.43 min in extracted RNA from 22Rv1s on the LoC device. Figure 5a illustrates the comparison between the LoC device and the benchtop assays for detection of AR-V7 and YAP1 mRNA. The LoC values are largely comparable to the pH change and TTP values of the benchtop assay, indicating that the LoC device is a robust method for AR-V7 and YAP1 detection in PCa cell lines. YAP1 mRNA detection occurs in 8.01 ± 0.64 min with DU145 mRNA and 13.22 ± 1.59 min with 22RV1 mRNA. The change in TTP values between the two PCa cell lines on average is 5.22 min, which is increased relative to the benchtop assay. As such, it provides a greater distinction between YAP1 mRNA concentrations within 22Rv1 and DU145 cell lines.

## Discussion

This paper presents a foundation for the LoC detection of circulating mRNA in PCa. The two novel assays judiciously developed for this work are, to the authors’ knowledge, the first RT-qLAMP experiments for the detection of AR-V7 and YAP1 mRNA. Authentication of AR-V7 and YAP1 detection was confirmed with extracted RNA from PCa cell lines and RT-qPCR. These RT-pHLAMP assays produced a suitable pH change for use with complementary metal oxide semiconductor technology containing an array of ISFET sensors. This compatibility resulted in a LoC device with potential for direct PoC usage. Detection of synthetic RNA was achieved at a sensitivity of 3 × 10^1^ copies per reaction for both markers. The RT-pHLAMP reactions, on account of their isothermal nature, remove the necessity of specialized and expensive thermal cycling equipment required for RT-qPCR experiments. Further development of these assays for the detection of circulating mRNA directly in serum would further increase their potential for rapid prognostics.

The PROPHECY study, completed in 2019, has confirmed that the presence of circulating AR-V7 mRNA associates with a lower progression-free survival and overall survival in mCRPC patients treated with enzalutamide and abiraterone.^12^ This illustrates that the presence of circulating AR-V7 mRNA could be used to monitor mCRPC patients and direct treatment options. Therefore, PoC detection of AR-V7 through this novel assay could show clinical benefit to mCRPC patients.

YAP1 concentration can be distinguished in the quantitative RT-pHLAMP assay and detected using the LoC device with varying TTP values in two PCa cell lines. High YAP1 concentration can be illustrative of PCa tumors progressing to EMT, whereas low YAP1 concentration could indicate advancement of mCRPC toward docetaxel resistance. In conjunction, AR-V7 and YAP1 mRNA detection on a LoC device could result in clinically actionable information, obtainable rapidly (<20 min), sensitively, and directly in the clinic. Further evaluation utilizing blood samples from PCa patients will be required to confirm the validity of these assays for use directly in hospitals. Progression in sample preparation allowing for direct detection of circulating markers in the blood in RT-pHLAMP reactions will expedite the time taken from biofluid extraction to a prognosis using this PoC device. Optimization of plasma-based reactions on the ISFET biosensor will be crucial to determine appropriate loading parameters for clinical samples. Alternatively, a rapid RNA extraction technique coupled to the LoC device could remove the necessity of direct testing in plasma.

Further detection of a larger range of circulating nucleic acid biomarkers could create a multiplex LoC device to serve as a prognostic test to personalize medication for PCa patients. The development of more RT-pHLAMP assays in conjunction with the ISFET LoC device could result in a robust handheld device for rapid, reliable, and simultaneous detection of multiple circulating prognostic PCa biomarkers.

## Materials and Methods

### Synthesis of Synthetic RNA Targets

RNA fragments of AR-V7 and YAP1 sequences were synthesized from DNA gBlocks (Integrated DNA Technologies) utilizing the HiScribe T7 Quick High Yield RNA Synthesis Kit (NEB) according to the manufacturer’s instructions including the DNase step. Stock concentrations were maintained at 3 × 10^10^ copies per μL and stored at −80 °C in preparation for experiments.

### RT-qLAMP Experiments

All reactions were completed in triplicate. Each 10 μL experiment contained: 1 μL 10× isothermal buffer [New England Biolabs (NEB)], 0.6 μL of MgSO_4_ (100 mM stock), 1.4 μL of dNTPs (10 mM stock of each nucleotide), 0.6 μL of BSA (20 mg/mL stock), 0.8 μL of betaine (5 M stock), 0.25 μL of SYTO 9 green (20 μL stock), 0.25 μL of NaOH (0.2 M stock), 0.042 μL of Bst 2.0 DNA polymerase (120,000 U/mL stock, NEB), 0.1 μL of RiboLock RNAse Inhibitor (40 U/μL stock, Thermo Fisher), 0.3 μL of WarmStart RTx reverse transcriptase (15,000 U/mL, NEB), 1 μL of 10× LAMP primer mix (20 μM FIP and BIP, 10 μM LB and LF, 2.5 μM F3 and B3), 1 μL of RNA sample, and the remaining solution was topped up to 10 μL with nuclease-free water. Reactions were conducted at 63 °C for 35 min. One melting curve from 63 to 97 °C was conducted to confirm the specific amplification of the reaction at a ramp of 0.2 °C/s. Reactions were conducted with a LightCycler 96 instrument (Roche Diagnostics) in 96-well plates.

### RT-pHLAMP Experiments

All reactions were completed in triplicate. Each 10 μL experiment contained: 1 μL of customized isothermal buffer, 0.5 μL of MgSO_4_ (100 mM stock), 1.4 μL of dNTPs (10 mM stock of each nucleotide), 0.6 μL of BSA (20 mg/mL stock), 0.25 μL of SYTO 9 green (20 μM stock), 0.25 μL of NaOH (0.2 M stock), 0.042 μL of Bst 2.0 WarmStart DNA polymerase (120,000 U/mL stock, NEB), 0.3 μL of WarmStart RTx reverse transcriptase (15,000 U/mL stock, NEB), 1 μL of 10× LAMP primer mix (20 μM FIP and BIP, 10 μM LB and LF, 2.5 μM F3 and B3), 1 μL of RNA sample, and the remaining solution was topped up to 10 μL with nuclease-free water. For plasma experiments, the RNA sample was serially diluted in TE buffer. 10 μL of citrated human plasma (TCS Biosciences) was spiked with 1 μL of the RNA sample. 1 μL of this solution was then added to each reaction. Reactions were conducted at 63 °C for 35 min. One melting curve from 63 to 97 °C was conducted to confirm the specific amplification of the reaction at a ramp of 0.2 °C/s. Reactions were conducted with a LightCycler 96 instrument (Roche Diagnostics) in 96-well plates. Reactions were scaled up to either 12 or 20 μL reactions for implementation onto the LoC device and proportions of each reagent were kept the same.

### RT-qPCR Experiments

All reactions were completed in triplicate. RT-qPCR reactions were completed in two steps. 50 ng of mRNA samples were initially converted to cDNA with a RevertAid First Strand cDNA synthesis kit (Thermo Fisher Scientific) as per the manufacturer’s instructions including the optional step for GC rich regions. cDNA was used immediately for qPCR assays. qPCR experiments were conducted in 10 μL quantities and contained the following: 5 μL of Fast SYBR Green Master Mix (Applied Biosystems), 2 μL of cDNA sample, 0.5 μL of forward primer (250 nM, 5 μM stock), 0.5 μL of reverse primer (250 nM, 5 μM stock), and nuclease-free water was added to make the reaction volume up to 10 μL. Reactions were aliquoted into a 96-well plate for analysis with a StepOnePlus Real-Time PCR system (Applied Biosystems). Reactions were initially heated to 95 °C for 20 s. The cycling stage included heating at 95 °C for 3 s followed by 60 °C for 30 s. The cycling stage was repeated for 40 cycles. Melting curves were conducted with heating to 95 °C for 15 s followed by 60 °C for 1 min.

### Translation of RT-pHLAMP onto the Lab-On-Chip Device

The LoC system detects changes in proton concentration on the interface of the RT-pHLAMP assay solution with the passivation layer (Si_3_N_4_). The ISFET array is composed of 56 × 78 ISFET pixels (4368 individual sensors, 2 × 4 mm).^49^ Temperature was maintained at 63 °C with a Peltier heating module contacting the underside of the cartridge. The LoC device was battery-powered and data were sent to an android phone through a Bluetooth connection. Data extracted from the mobile phone was run through a MATLAB (R2021b) algorithm designed to spot for amplification events. The RT-pHLAMP assay solutions were housed in an acrylic manifold with either 12 or 20 μL sized chambers. Adhesive gaskets is composed of Tesa double-sided smooth lamination filmic tape that sealed the acrylic manifold to the cartridge. A 0.03 mm chlorodized silver wire served as the Ag/AgCl reference electrode. This electrode was in contact with the assay solution and was placed between the adhesive gasket and the microchip’s surface. Nuclease-free water was added to the chamber of the manifold for the first 700 s to equilibrate the system and set a common voltage across the ISFET array. The water was then extracted and the RT-pHLAMP reaction mixture was added. All samples that contained synthetic RNA or extracted RNA were run for 30 min after the addition of the RT-pHLAMP reaction. Negative controls contained nuclease-free water instead of RNA and were run for 20 min after the addition of the RT-pHLAMP reaction. All reactions were completed in triplicate. Confirmation of amplification presence in these reactions was confirmed with a Qubit 3.0 fluorometer (Invitrogen). Measured pH values post-reaction were conducted with a microFET pH probe (Sentron).

### RNA Extraction from Prostate Cancer Cell Lines

22Rv1 and DU145 cell lines were cultured in T-75 flasks with RPMI-1640 containing FBS (10%) and l-glutamine (5 mM). Cells were passaged at 70% confluency to maintain optimal growth and kept to under 10 passages post-thawing. For RNA extraction, cells were harvested at 70% confluency and spun down to remove media. For the 22Rv1 cells, RNA extraction was performed using the Total RNA Miniprep Kit (Monarch) as per manufacturer’s instructions including the DNase I digestion step. For DU145 cells, RNA extraction was performed using the RNeasy Mini Extraction kit (Qiagen) as per the manufacturer’s instructions. In both cases, RNA was eluted in 50 μL and RNA quantity/quality was measured using a Nanodrop D1000. Extracted RNA was stored at −80 °C until use.

Drosophila Schneider 2 cells were grown in Schneider’s Insect Medium with FBS (10%) and penicillin–streptomycin (1%) in T-75 flasks at room temperature. RNA extraction was performed using TRIzol LS Reagent (Thermo Fisher) according to the manufacturer’s instructions. RNA quantity/quality was measured using a Nanodrop D1000. Extracted RNA was stored at −80 °C until use.

### Statistical Analyses

Welch’s *t*-test was chosen to determine the statistical significance of YAP1 RT-pHLAMP TTP values and YAP1 qPCR Cq values in the 22Rv1 and DU145 cell lines experiments. This test is commonly utilized in scenarios where the two compared data sets have different variance or different sample sizes.^50^ The null hypothesis in each case was that the mean TTP or *C*_q_ value was the same between the 22Rv1 and DU145 prostate cancer cell lines.

The calculation for degrees of freedom (*v*) for Welch’s *t*-test is shown below (eq 1), where *s*_1_ and *s*_2_ are the standard deviations of the two data sets and *N*_1_ and *N*_2_ are the number of samples per data set.
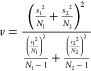
1

The equation for *t* value for Welch’s *t*-test of unequal variance is shown below (eq 2), where  and  are the mean values of the two data sets.
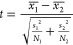
2

The null hypothesis was rejected when *p* < 0.05.

## Supporting Information Available

Additional experimental details, RT-LAMP and PCR primer sequences, and further mathematical equations (PDF)
