# Supplementary material for: Detection of YAP1 and AR-V7 mRNA for Prostate Cancer Prognosis Using an ISFET Lab-On-Chip Platform
Source: ACS Sens. 2022 Nov 11;7(11):3389–98. doi: 10.1021/acssensors.2c01463 (PMC9706784; doi:10.1021/acssensors.2c01463)
Supplement: Supplementary file 1 — se2c01463_si_001.pdf [file se2c01463_si_001.pdf]

Supplementary information:

Detection of YAP1 and AR-V7 mRNA for Prostate Cancer prognosis using an ISFET Lab-On-Chip platform

Joseph Broomfield [1,2], Melpomeni Kalofonou [1], Thomas Pataillot-Meakin [2,3,4], Sue M. Powell [2] , Rayzel C. Fernandes [2], Nicolas Moser [1], Charlotte L. Bevan [2] and Pantelis Georgiou [1,*].

[1] Centre for Bio-Inspired Technology, Department of Electrical and Electronic Engineering, Imperial College London, London, SW7 2AZ, United Kingdom.

[2] Imperial Centre for Translational and Experimental Medicine, Department of Surgery and Cancer, Imperial College London, London, W12 0NN, United Kingdom.

[3] Molecular Science Research Hub, Department of Chemistry, Imperial College London, London, W12 0BZ, United Kingdom.

[4] Sir Michael Uren Hub, Department of Bioengineering, Imperial College London, London, W12 0BZ, United Kingdom.

[*] Corresponding author. Email: pantelis@imperial.ac.uk

Contents

1 DNA gblocks **2**
1.1 AR-V7 gblock 2
1.2 YAP1 gblock 3
1.3 AR-FL gblock 3

2 AR-FL primers **3**

3 Primer optimisation experiments **4**
3.1 AR-V7 4
3.2 YAP1 5
3.3 AR-FL 6

4 AR-V7 specificity **7**

5 qPCR data for AR-V7 mRNA in PCa cell lines **9**

6 qPCR data for YAP1 mRNA in PCa cell lines **10**

7 qPCR primer sequences **10**

8 AR-V7 and YAP1 melting curve analysis **11**

9 Synthetic YAP1 RNA reactions with plasma and off-target RNA **12**

10 Cq value determination **12**

11 Mathematical equations **12**

1 DNA gblocks

Each gblock (Integrated DNA Technologies) contains the 5'-TAATACGACTCACTATAGG-3' promoter region for T7 polymerase. These gblocks were transcribed into RNA for use in synthetic RNA experiments as described under the Materials and Methods section.

1.1 AR-V7 gblock

Accession number: NM_001348061
5'-TAATACGACTCACTATAGGGGACTCAAGGTGTCACCTTGGACAAGAAG
CAACTGTGTCTGTCTGAGGTTCCTGTGGCCATCTTTATTTGTGTATTAGGC
AATTCGTATTTCCCCCTTAGGTTCTAGCCTTCTGGATCCCAGCCAGTGACC
TAGATCTTAGCCTCAGGCCCTGTCACTGAGCTGAAGGTAGTAGCTGATCCA
CAGAAGTTCAGTAAACAAGGACCAGATTTCTGCTTCTCCAGGAGAAGAAGC
CAGCCAACCCCTCTCTTCAAACACACTGAGAGACTACAGTCCGACTTTCCC

TCTTACATCTAGCCTTACTGTAGCCACACTCCTTGATTGCTCTCTCACATC
ACATGCTTCTCTTCATCAGTTGTAAGCCTCTCATT-3'

1.2 YAP1 gblock

Accession number: NM_001130145
5'-TAATACGACTCACTATAGGGGTGCTGCCATTAAAGGCAGCTGTTCTAG
AGTTTCAGTCACCTAAGTACACCCACAAAACAATATGAATATGGAGATCTT
CCTTTACCCCTCAACTTTAATTTGCCCAGTTATACCTCAGTGTTGTAGCAG
TACTGTGATACCTGGCACAGTGCTTTGATCTTACGATGCCCTCTGTACTGA
CCTGAAGGAGACCTAAGAGTCCTTTCCCTTTTTGAGTTTGAATCATAGCCT
TGATGTGGTCTCTTGTTTTATGTCCTTGTTCCTAATGTAAAAGTGCT-
TAACTGCTTCTTGGTTGTA TTGGGTAGCATTGGGATAAGATTTTAACTGGG
TATT CTTGAATTGCTTTTAC-3'

1.3 AR-FL gblock

Accession number: NM_000044
5'-TAATACGACTCACTATAGGCTCCGTGCAGCCTATTGCGAGAGAGCTGCA
TCAGTTCACTTTTGACCTGCTAATCAAGTCACACATGGTGAGCGTGGACTTT
CCGGAAATGATGGCAGAGATCATCTCTGTGCAAGTGCCCAAGATCCTTTCTG
GGAAAGTCAAGCCCATCTATTTCCACACCCAGTGAAGCATTGGAAACCCTAT
TTCCCCACCCCAGCTCATGCCCCCTTTCAGATGTCTTCTGCCTGTTATAACT
CTGCACTACTCCTCTGCAGTGCCTTGGGGAATTTCCTCTATTGATGTACAGT
CTGTCATGAACATGTTCCTGAATTCTATTTGCTGGGCTTTTTTTTTCTCTTT
CTCTCCTTTCTTTTTCTT-3'

2 AR-FL primers

F3 - 5'-AGACTCTCTCCAGACAGC-3'
B3 - 5'-GACTTTAAGTTTTGGATTTGATCTG-3'
LF - 5'-TTCCAGGGCTATGCAGGGG-3'
LB - 5'-GTTTGACCCACTACAAGGGGT-3'
FIP - 5'-TTCGTAGACAGTCAGCCTCACTACCCGAGCATGGCCCC-3'
BIP - 5'- GCCAAGGGAGTGGTTTTTCCTGATTCCCATGAC-3'

3 Primer optimisation experiments

3.1 AR-V7

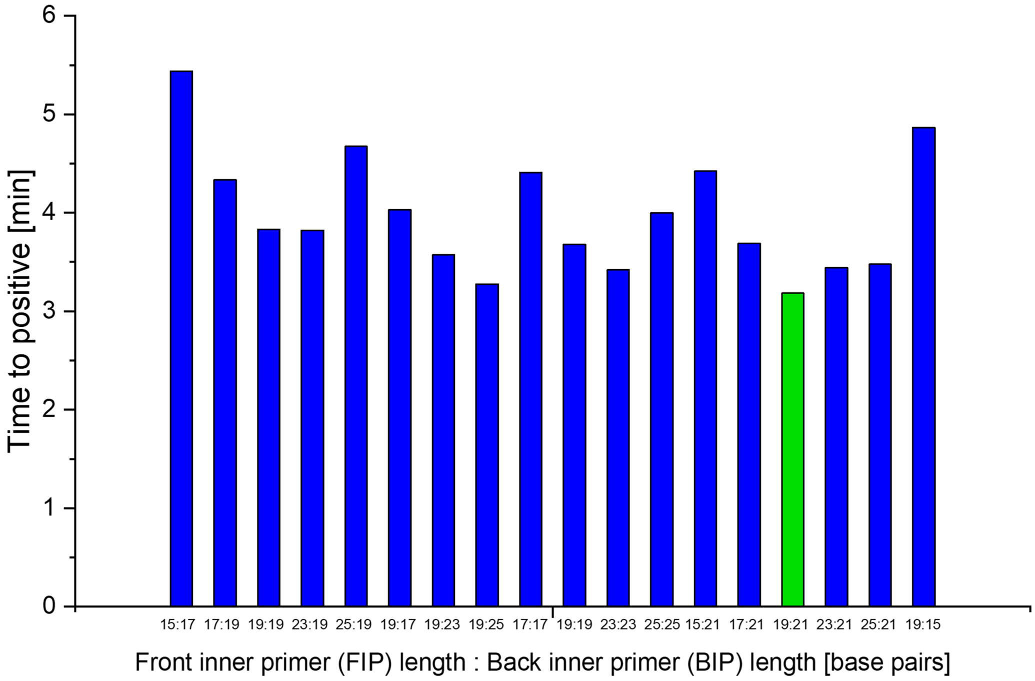

Figure S1: Optimisation RT-qLAMP experiment determining the fastest combination of FIP and BIP base pair values for the AR-V7 synthetic target. The fastest combination is shown in green and its sequence is shown in the main manuscript. 3 x 10^8 copies of synthetic RNA are detected in these reactions.

3.2 YAP1

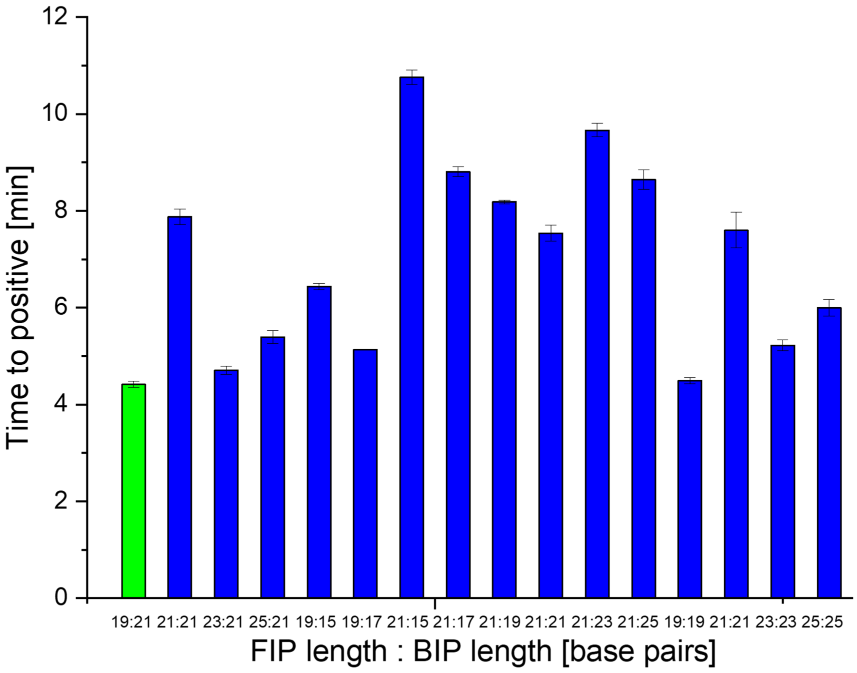

Figure S2: Optimisation RT-qLAMP experiment determining the fastest combination of FIP and BIP base pair values for the YAP1 synthetic target. The fastest combination is shown in green and its sequence is shown in the main manuscript. 3 x 10^8 copies of synthetic RNA are detected in these reactions.

3.3 AR-FL

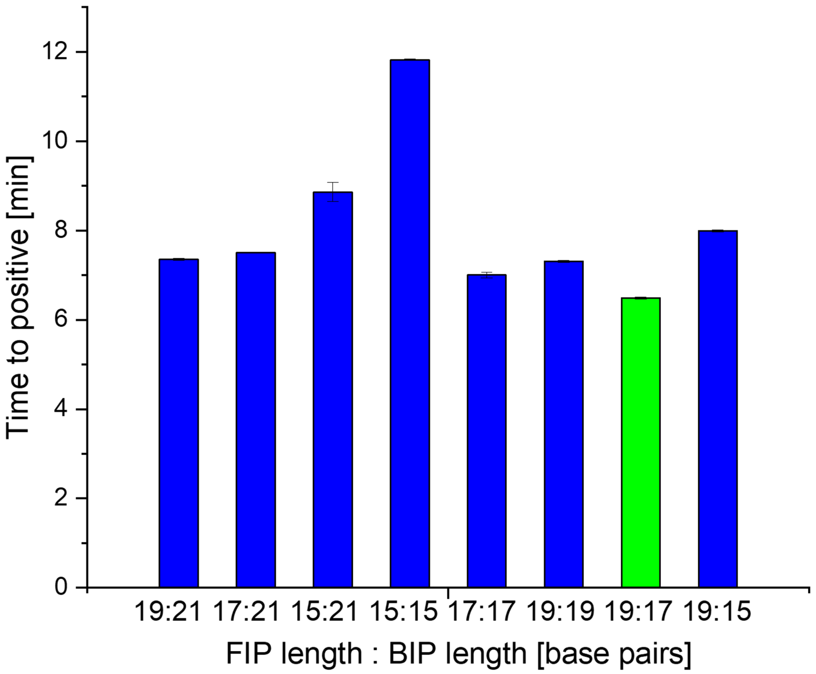

Figure S3: Optimisation RT-qLAMP experiment determining the fastest combination of FIP and BIP base pair values for the ARFL synthetic target. The fastest combination is shown in green and its sequence is shown above under AR-FL primers. 1 x 10^8 copies of synthetic RNA are detected in these reactions.

4 AR-V7 specificity

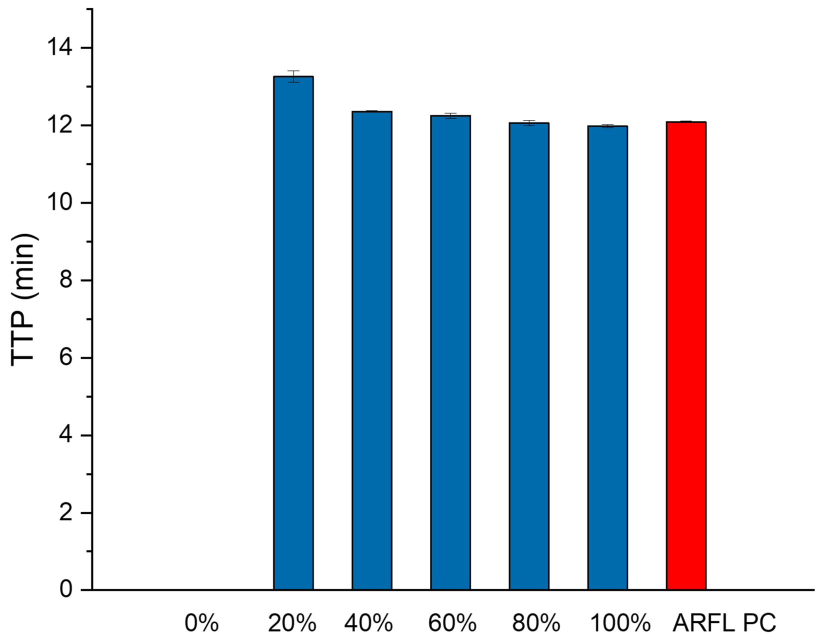

Figure S4: This graph shows the data of the AR-V7 RT-qLAMP assay spiked with AR-FL synthetic RNA. Percentages shown on the x axis indicate the relative quantity of AR-V7 synthetic RNA in the assay. The total RNA copies per reaction was 1 x10^5 copies. For example in the 20 % assay 2x10^4 copies of AR-V7 synthetic RNA were present and 8x10^4 copies of AR-FL synthetic RNA were present.

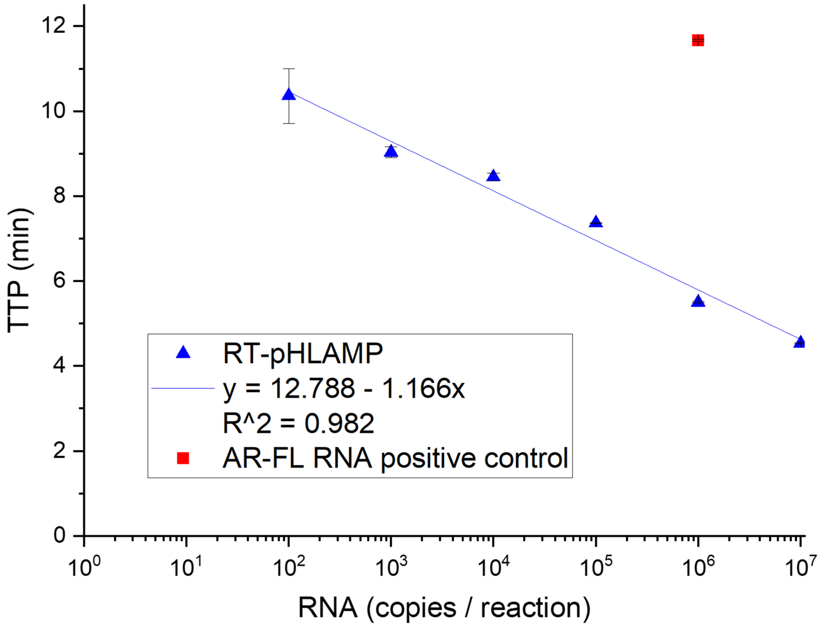

Figure S5: This graph shows the effect of synthetic AR-FL RNA presence on reducing the efficiency of the AR-V7 RT-pHLAMP assay. A positive control for AR-FL was included to confirm the presence of the RNA in these assays (shown in red).

5 qPCR data for AR-V7 mRNA in PCa cell lines

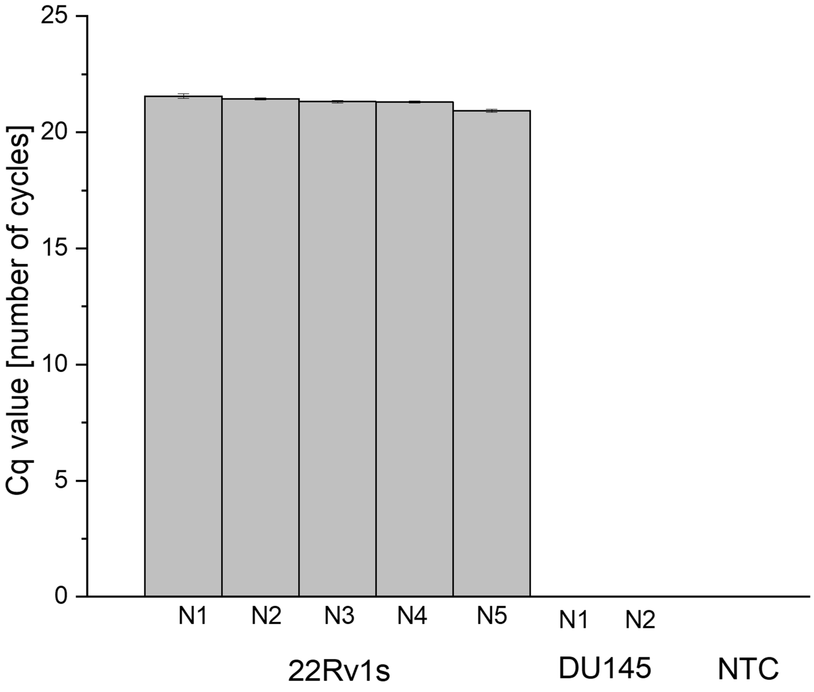

Figure S6: This shows the qPCR data in two prostate cancer cell lines for the presence of AR-V7. This assay was detecting cDNA which was reverse transcribed from extracted RNA from these cell lines. The same extracted RNA was used in the RT-pHLAMP reactions and Lab-on-Chip reactions in the main manuscript.

6 qPCR data for YAP1 mRNA in PCa cell lines

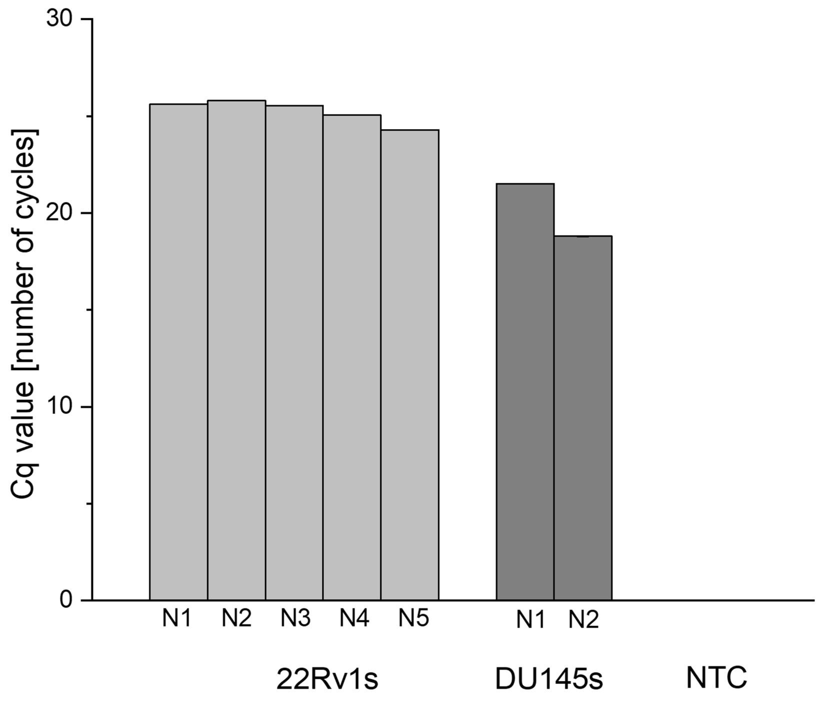

Figure S7: This shows the qPCR data in two prostate cancer cell lines for the presence of YAP1. This assay was detecting cDNA which was reverse transcribed from extracted RNA from these cell lines. The same extracted RNA was used in the RT-pHLAMP reactions and Lab-on-Chip reactions in the main manuscript.

7 qPCR primer sequences

YAP1 forward primer 5'- GCACCTCTGTGTTTTAAGGGTCT - 3'
YAP1 reverse primer 5' - CAACTTTTGCCCTCCTCCAA - 3'
AR-V7 forward primer 5' - GACTCTGGGAGAAAAATTCCG - 3'
AR-V7 reverse primer 5' - CTCCAGACTATCCACTAGAG - 3'

8 AR-V7 and YAP1 melting curve analysis

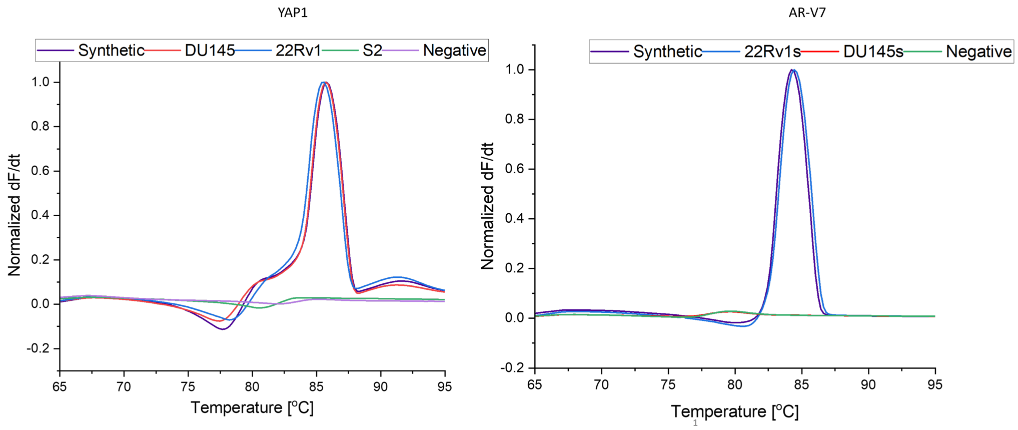

Figure S8: Melting curves from 65 ^oC to 95 ^oC for YAP1 and AR-V7 RT-pHLAMP experiments. Melting curves for synthetic RNA, RNA from extracted prostate cancer cell lines and RNA extracted from the S2 cell line is shown.

9 Synthetic YAP1 RNA reactions with plasma and off-target RNA

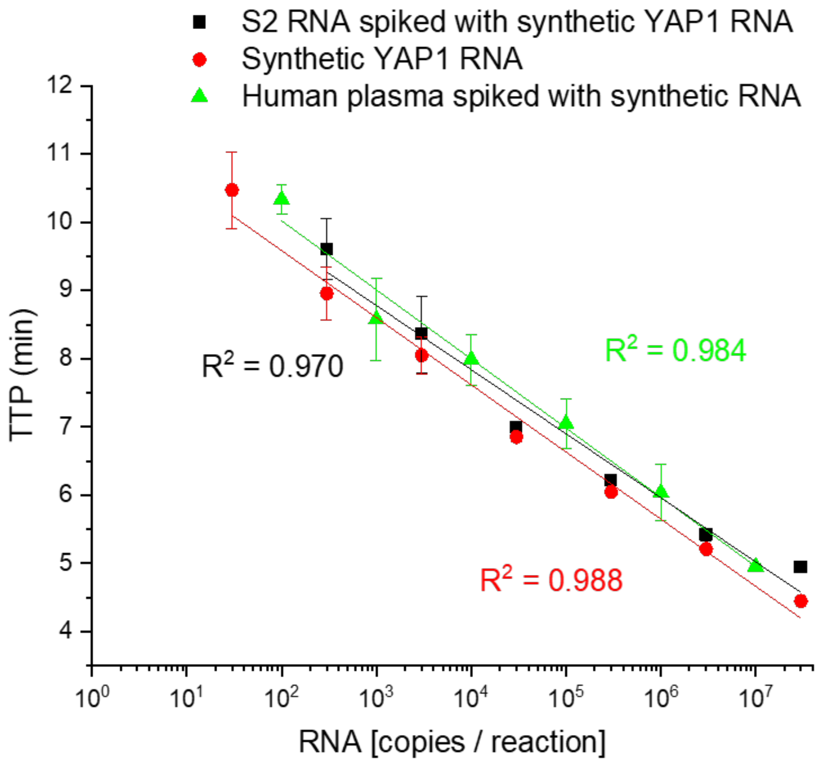

Figure S9: The relative time to positives for the detection of YAP1 synthetic RNA under different experimental conditions.

10 Cq value determination

Lightcycler 96® (Roche diagnostics) devices utilise a predefined threshold fluorescence to calculate Cq values for SYBR green and SYBR green- like dyes. The predefined threshold for fluorescence for these fluorophores is 0.2.

11 Mathematical equations

The conversion of chemical voltage output to pH is shown through the two equations below.

$$V_{chem} = \gamma + \alpha S_n pH \tag{S1}$$

$$[H^+] = 10^{-pH} \tag{S2}$$

In equation S1 V_{chem} is the chemical voltage, γ is a constant chemical term and α is the deviation from the Nernstian sensitivity (S_n) [1].

References

[1] Nicolas Moser et al. "ISFETs in CMOS and Emergent Trends in Instrumentation: A Review". In: *IEEE Sensors Journal* 16.17 (2016), pp. 6496–6514. ISSN: 1530437X. DOI: 10.1109/JSEN.2016.2585920.
